# Competitive co-adsorption of bacteriophage MS2 and natural organic matter onto multiwalled carbon nanotubes

**DOI:** 10.1016/j.wroa.2020.100058

**Published:** 2020-06-10

**Authors:** Céline Jacquin, Diya Yu, Michael Sander, Kamila W. Domagala, Jacqueline Traber, Eberhard Morgenroth, Timothy R. Julian

**Affiliations:** a Eawag, Swiss Federal Institute of Aquatic Science and Technology, 8600, Dübendorf, Switzerland; b Institute of Biogeochemistry and Pollutant Dynamics (IBP), Department of Environmental Systems Science, ETH Zürich, 8092, Zürich, Switzerland; c Empa, Swiss Federal Laboratories for Materials Science and Technology, Laboratory for High Performance Ceramics, Überlandstrasse 129, 8600, Dübendorf, Switzerland; d AGH, University of Science and Technology, Faculty of Materials Science and Ceramics, al. Mickiewicza 30, 30-059, Krakow, Poland; e ETH Zürich, Institute of Environmental Engineering, 8093, Zürich, Switzerland; f Swiss Tropical and Public Health, P.O. Box, 4001, Basel, Switzerland; g University of Basel, P.O. Box, 4002, Basel, Switzerland

**Keywords:** Multiwalled carbon nanotubes, Virus treatment, Natural organic matter, Competitive adsorption, Drinking water

## Abstract

A leading challenge in drinking water treatment is to remove small-sized viruses from the water in a simple and efficient manner. Multi-walled carbon nanotubes (MWCNT) are new generation adsorbents with previously demonstrated potential as filter media to improve virus removal. This study therefore aimed to evaluate the field applicability of MWCNT-filters for virus removal in water containing natural organic matter (NOM) as co-solute to viruses, using batch equilibrium experiments. Contrary to previous studies, our results showed with MS2 bacteriophages single-solute systems that the affinity of MWCNT for MS2 was low, since after 3 h of equilibration only 4 log_10_ reduction value (LRV) of MS2 (20 mL at an initial concentration of 10^6^ PFU MS2/mL) were reached. Single solute experiments with Suwannee river NOM (SRNOM) performed with environmentally-relevant concentrations showed MWCNT surface saturation at initial SRNOM concentrations between 10 and 15 mgC/L, for water pH between 5.2 and 8.7. These results suggested that at NOM:virus ratios found in natural waters, the NOM would competitively suppress virus adsorption onto MWCNT, even at low NOM concentrations. We confirmed this expectation with SRNOM-MS2 co-solute experiments, which showed an exponential decrease of the MS2 LRV by MWCNT with an increase in the initial SRNOM concentration. More interestingly, we showed that pre-equilibrating MWCNT with a SRNOM solution at a concentration as low as 0.4 mgC/L resulted in a LRV decrease of 3 for MS2, due to the formation of a negatively charged SRNOM adlayer on the MWCNT surface. Complementary batch experiments with natural NOM-containing waters and competition experiments with SRNOM in the presence of CaCl_2_ confirmed that the presence of NOM in waters challenges virus removal by MWCNT-filters, irrespective of the concentration and type of NOM and also in the presence of Ca^2+^. We therefore conclude that MWCNT-filters produced with commercially available pristine MWCNT cannot be considered as a viable technology for drinking water virus removal.

## Highlights

• LRV of MS2 increase with MWCNT mass, but are little affected by solution pH.
• SRNOM adsorption onto MWCNT is pH dependent.
• SRNOM competes with MS2 for adsorption to MWCNT, even at low SRNOM concentrations.
• NOM suppresses MS2 adsorption, irrespective Ca2+ concentration and NOM composition.
• In practice, MWCNT is insufficiently selective for viruses to ensure virus removal.

## Graphical abstract

**Figure:**
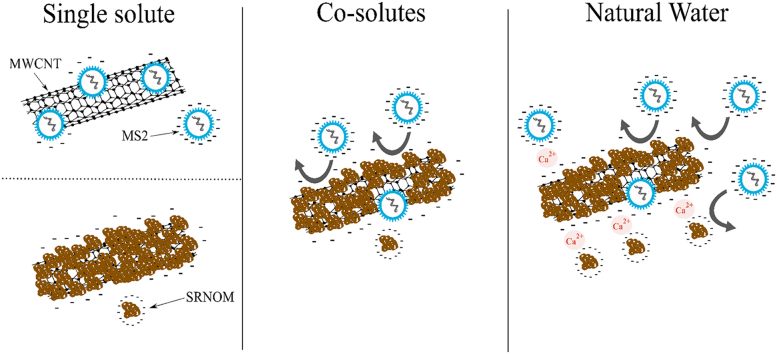

## 1 Introduction

Viruses are responsible for a large share of the global burden of respiratory and diarrheal infectious diseases (Kotloff et al., 2012) and can be responsible for major outbreaks. One major challenge to reduce mortality from waterborne viruses is the efficient removal of viruses in drinking water in low and middle-income countries. A promising strategy to prevent virus waterborne diseases are point-of-use (POU) treatments deployed in households and within communities. In these decentralized water facilities, water treatment technologies selected have low costs and maintenance, but are sustainable and easy to use (Peter-Varbanets et al., 2009). However, many conventional water treatment processes implemented in the context of POU may have minimal impact on virus removal or inactivation. For example, microfiltration and ultrafiltration is largely ineffective in removing viruses, due to their small size (Tanneru et al., 2013). To meet the local operation requirements and an adequate virus removal, new processes urgently need to be developed (Rahaman et al., 2012).

Carbon nanotubes (CNT) are broadly considered promising materials for future water treatment applications, including filtration and/or adsorption. The unique features of CNT include high surface areas, hydrophobicity, porosity, rapid adsorption kinetics, simple regeneration techniques and good mechanical and thermal stability. As a result, CNT are promising alternatives to traditional adsorbents (Elsehly et al., 2018; Sarkar et al., 2018; Shimizu et al., 2018). Since the invention of CNT, scientists have shown that these materials have antimicrobial properties, can be used for removal of heavy metals and organic pollutants by complexation and adsorption, respectively (Sun et al., 2012; Smith and Rodrigues, 2015). Studies have further suggested that CNT are also capable to remove viruses. Brady-Estévez et al. (2010b) demonstrated that a filter composed of a mixture of single-walled carbon nanotubes (SWCNT) and multi-walled carbon nanotubes (MWCNT) reached a virus log_10_ removal value (LRV) of 6 for MS2, PRD1 and T4 bacteriophages, at a flux through the filter of 160 L/m^2^/h. In another study, MS2 removal by a MWCNT filter was between 5 and 8 LRV at low pressure and LRV was higher than what was achieved using a SWCNT filter (Brady-Estévez et al., 2010a, 2010c). The higher removal observed for MWCNT filters was ascribed to physical retention of MS2 by the entangled CNT network that was deposited onto the filter in combination with MS2 adsorption onto MWCNT. Numerous virus-sorbent interaction forces are expected to drive MS2 adsorption onto MWCNT. Given that MWCNT are largely apolar (‘hydrophobic’), they tend to form aggregates and bundles with apolar internal cavities to the surface of which MS2 may adsorb by the hydrophobic effect (Chandler, 2005; Armanious et al., 2016a). In addition, van der Waals (VdW) forces, which operate at short virus-surface separation distances, contribute to MS2-MWCNT interactions. Overall, the past work supports the potential of MWCNT filters to complement existing water purification technologies that are already used in low and middle-income countries for households or water kiosks drinking water treatment.

Under field conditions, however, natural water sources contain particles in suspension and colloids (>45 μm) that can affect the efficiency of the MWCNT-filters by clogging the filter. Applying a first pre-filtration step, like gravity-driven membrane (GDM) filtration, would eliminate the potential interference from suspended particles (Pronk et al., 2019). However, this pre-filtration step is not removing natural organic matter (NOM), which is smaller than virions. NOM is ubiquitous in water and has also a high affinity to MWCNT surfaces (Hyung and Kim, 2008; Ateia et al., 2017; Shimizu et al., 2018). The high affinity of NOM for MWCNT might be a drawback for virus removal by MWCNT-filters, as observed Brady-Estévez et al. (2010c). Using a MWCNT-filter, the authors observed that MS2 LRV by MWCNT filter decreased from 5–6 to 0.17–1 in presence of SRNOM or alginate at concentrations higher than 1 mg/L. The authors hypothesized that NOM likely competed with the virus for adsorption sites in the MWCNT surface. It is likely that the decrease in MS2 LRV in the presence of NOM was due to the formation of a negatively charged NOM adlayer that electrostatically repelled MS2 from the surface, given that MS2 is negatively charged at circumneutral pH (i.e., isoelectric point of the virus (IEP = 3.9)). Armanious et al. (2016b) used quartz-crystal microbalance with dissipation (QCM-D) monitoring to directly demonstrate electrostatic repulsion of MS2 from NOM adlayers that formed on a positively charged surface (self-assembled monolayers of alkyl-thiols formed from ethanolic solution of cysteamine (SAM-NH_3_^+^)) at pH > IEP of MS2. Nonetheless, Brady-Estévez et al. (2010c) reported that NOM supposedly had a negligible negative effect on virus removal by MWCNT-filter when treating water samples with NOM concentrations below 1 mg/L. The authors concluded that MWCNT-filter technology was a cost-effective technology for point-of-use virus removal in low NOM waters. The study therefore assumed that there is a threshold concentration of NOM below which virus removal by MWCNT filters remains unaffected. However, the threshold observed in this study could also be explained by a short contact time of the NOM with the filter surfaces during filtration, resulting in a limited co-adsorption effect at low NOM concentrations. Indeed, Armanious et al. (2014) showed using QCM-D adsorption studies that continuously supplying NOM, even at very low concentrations, to positively charged adsorbent surface resulted in NOM adsorption until the entire adsorbent surface was saturated. Armanious et al. (2014) questions the notion of a critical NOM threshold concentration in solution below which virus removal from the same solution is not affected, as suggested by Brady-Estévez et al. (2010c). Another important aspect in assessing the applicability of MWCNT-filters as an efficient and innovative solution for point-of-use virus removal is to study potential effects of the ionic composition of the solution on MS2 removal and NOM competition. Brady-Estévez et al. (2010c) and Brady-Estévez et al. (2008) showed that increasing the ionic strength (IS) by adding NaCl, increased MS2 removal by CNT-filters due to the suppression of repulsive electrostatic forces between viruses and CNT. Other studies showed that divalent and trivalent cations could increase the adsorption of MS2 to surfaces. Pumpens (2020) reported that MS2 was found to adsorb on anionic clay-like nanocomposites together with Zn^2+^, Mg^2+^ and Al^3+^. Higher adsorption of MS2 to sandy loam soil in presence of oxidized metal ions like ferric oxihydroxides was noted by Witzany (2010). Farrah (1982) showed that the presence of magnesium increased MS2 adsorption to membrane filters, while Brady-Estévez et al. (2010c) showed that it decreased the virus removal by MWCT-filters. Finally, Brady-Estévez et al. (2010c) showed that adding CaCl_2_ improved MS2 virus removal by MWCNT-filters in presence of NOM. NOM competitive effect mitigation during virus removal by MWCNT-filters in presence of Ca^2+^ may result from Ca^2+^ forming cationic bridges between negatively charged carboxylate and phenolate groups in the NOM adlayer and the negatively charged amino acids on the MS2 virion surface. The cation bridges formation would increase MS2 adsorption through the formation of NOM-Ca^2+^-MS2 complexes, as compared to Ca^2+^-free solutions, thereby overcoming direct MS2-NOM electrostatic repulsion (Kalinichev et al., 2011; Kloster et al., 2013). Evidence in support of cation bridges was also reported by Pham et al. (2009), who showed higher attachment efficiency of MS2 onto the SRNOM-adlayers in presence of Ca^2+^. Amongst all ions present in natural waters, the competition mitigation effect observed in presence of Ca^2+^ is of major interest since it might allow using MWCNT-filters to remove viruses from natural waters containing high calcium concentrations. However, there is a research gap concerning the adsorption mechanisms of MS2 onto MWCNT and the effect of NOM as co-solute and the presence of Ca^2+^ to critically evaluate the field application of MWCNT-filters for virus removal.

The goal of our work was to experimentally assess the competitive co-adsorption between MS2 bacteriophages and NOM to MWCNT. This information is critical to inform the feasibility of using MWCNT as adsorbent to produce filters to remove virus from waters that contain different types and concentrations of NOM and/or ionic compositions. To do so, we studied adsorption of MS2 bacteriophages to MWCNT in batch reactors, both in the absence and presence of NOM as co-adsorbate. MS2 bacteriophages were used as a surrogate for apolar and negatively charged enteric viruses of human health concern, such as Adenovirus (Shi et al., 2016). Furthermore, MS2 bacteriophage is a virus surrogate recommended by the World Health Organization (WHO) to evaluate virus removal of household water treatment options (World Health Organization, 2011) and it was used in previous studies investigating the virus removal by CNT-filters, allowing for better comparability between studies (Brady-Estévez et al., 2010b, 2010c). Briefly, batch experiments with MS2 were performed to estimate the minimum mass of MWCNT required to reach at least an MS2 LRV of 4, in line with the recommendation of the U.S. Environmental Protection Agency (US EPA, 2015). Subsequently, we performed single solute batch adsorption experiments with MS2 and SRNOM to evaluate at which concentrations these two solutes saturated the MWCNT surface. This information is critical to assess competitive effects in co-solute systems. Then, we assessed competitive co-adsorption to MWCNT in batch adsorption experiments containing both MS2 and SRNOM at different concentration ratios. Finally, for a more environmentally realistic assessment of the potential of MWCNT filters for virus removal, we used natural waters in batch experiments and determined the effect of Ca^2+^ on MS2 removal in SRNOM-Ca^2+^ batch experiments. The results from this work inform on the efficacy of MWCNT to produce virus-filters, a necessary first step toward application in the field for drinking water treatment.

## 2 Material and methods

### Multiwalled carbon nanotubes

MWCNT were purchased from CheapTubes (LOT number 180320; USA). The MWCNT had a specific surface area of 117 m^2^/g. Their length and outer diameter were equal to 10–30 μm and 20–30 nm, respectively. Metallic impurities in MWCNT were determined by ICP-MS after digestion of the MWCNT for 2 h in HNO_3_ (65%) and H_2_O_2_. Details about metal impurities are provided in the supplementary information (Table S1).

Prior to use MWCNT in batch adsorption experiments, MWCNT were bath-sonicated in ethanol (i.e., 100 mg MWCNT in 20 mL ethanol; 35 Hz; 120 W; 5min). The resulting suspension was subsequently vacuum-filtered onto 0.1 μm hydrophilic PVDF disc membranes (Durapore Membrane, Merck Millipore, Germany) and washed with 1.6 L of NanoPure Water (Milli-Q, Millipore, USA) to remove ethanol. The MWCNT deposited on the filter membrane were then collected and dried overnight at 60 °C before use.

### NOM and calcium solution preparation and collection of natural water samples

Suwannee river natural organic matter (SRNOM, 2R101N, RO isolation) served as model NOM and was purchased from the International Humic Substances Society (IHSS). SRNOM stock solution was prepared by adding 0.05 g of SRNOM into 500 mL of buffer solution (0.78 g/L NaH_2_PO_4_ and 0.58 g/L NaCl, IS = 26 mM, pH = 4.75), which was used as a dilution buffer for all batch experiments, and stirred overnight to ensure complete dissolution of SRNOM. The pH of the SRNOM stock solution was then adjusted to the targeted pH required (i.e., pH 5.2, 7.7 and 8.7) by adding small volumes of 5 M NaOH. The dissolved organic carbon (DOC) of the resulting SRNOM solutions was quantified using a TOC-L total carbon analyzer (Shimadzu, Japan).

Calcium stock solutions were prepared dissolving CaCl_2_ in buffer solution to obtain three different Ca^2+^ concentrations: 0.1 mmol/L, 1.9 mmol/L and 7.5 mmol/L, respectively.

To demonstrate the transferability of the results obtained with the model NOM to NOM in natural water samples, we also determined MS2 adsorption to MWCNT in water samples that we collected from four different sources: tap water, ground water, river water and wastewater treatment plant secondary effluent. We collected these water samples at Eawag (Dübendorf, Switzerland). More specifically, river and ground water were collected from the sampling stations of Chriesbach river and the local aquifer, respectively. The secondary effluent was sampled from Eawag’s pilot-scale wastewater treatment plant outlet. After collection, the samples were filtered through sterile 0.45 μm PES filters (Sartorius, Germany) to remove particulates before using the water samples in batch adsorption experiments. DOC and Ca^2+^ concentrations of these waters were quantified by TOC-L total carbon analyzer (Shimadzu, Japan) and ion chromatography (Metrohm 930 Compact IC Flex), respectively.

### Batch experiments

Adsorption of MS2 and NOM onto MWCNT was studied both in single solute and co-solute batch reactor setups and performed in duplicate. All batch reactor experiments were set up by weighing a known mass of MWCNT in 50 mL carbon-free glass vials (muffled at 450 °C for 4 h) followed by adding the targeted solution (20 mL), as shown in Table 1. pH values and DOC/Ca^2+^ concentrations used in our study are based on a literature review we have made from 85 studies that reported water chemistry characteristics of natural water samples from 42 countries. The data from the literature review were used to calculate average, upper and lower values (5th and 95th percentiles), thereby capturing the range of values found in natural waters that can be used as a drinking water source. The references of these studies are available in supplementary information (Section 4). Based on the results of adsorption kinetic experiments (Fig. S1 and Fig. S2 in supplementary information), the batch equilibration time was set to 3 h.

**Table 1:** Detailed description of different types of conducted adsorption experiments.

Effect of	pH	Mass of MWCNT (mg)	Concentration of MS2 (PFU/mL)	NOM type and concentration	Concentration of Ca^2+^ (mmol/L)	Analysis
MWCNT mass on MS2 log_10_ removal	5.2, 7.7 and 8.7	2.5 to 15	10^6^	–	–	MS2 PFU assay
SRNOM initial concentration on SRNOM adsorption	5.2, 7.7 and 8.7	15	–	SRNOM; from 2.5 to 25 mgC/L	–	DOC analysis and liquid chromatography coupled with organic carbon and organic nitrogen detectors (LC-OCD) analysis
initial MS2 concentration on MS2 log_10_ removal	7.7	15	Increased from 10^5^ to 10^9^	–	–	MS2 PFU assay
SRNOM as co-solute on MS2 log_10_ removal	7.7	15 and 5	10^6^	SRNOM; from 0 to 15 mgC/L	–	MS2 PFU assay
an SRNOM adlayer on MS2 log_10_ removal	7.7	15	10^6^ (added after 3 h of adsorption with SRNOM)	SRNOM; preloading from 0 to 15 mgC/L for 3 h	–	MS2 PFU assay
Ca^2+^ concentration on MS2 log_10_ removal in SRNOM co-solute systems	7.7	15	Fixed at 10^6^	SRNOM; 5 mgC/L	0.1, 1.9 and 7.5	MS2 PFU assay
NOM in natural water samples on MS2 log_10_ removal	7.7	15	Fixed at 10^6^	Tap water, ground water, river water and water from WWTP secondary effluent	Actual concentration of water samples	MS2 PFU assay and LC-OCD-OND analysis

After 3 h mixing at 40 rpm with a rotating mixer at room temperature, the samples were filtered through sterile 0.45 μm PES filters syringe (Sartorius, Germany) and the filtrate was further analyzed. For batch experiments aiming to study virus removal by MWCNT, with and without NOM co-solutes, a blank reactor with MS2 solution was included to determine MS2 concentrations after 3 h mixing. This blank was used to determine the concentration of virus available for adsorption onto MWCNT (C_i_) and thus accounted for any potential dilution variability, decay and adsorption to the glass vial during the batch experiment (decreases in the blanks were between 9 and 66.5%, or between 9∗10^4^ and 7∗10^5^ PFU/mL, depending on the experiment).

A reactor with 15 mg MWCNT in buffer solution was also ran as a blank for single solute SRNOM adsorption experiments, in order to quantify the amount of DOC that leached from the MWCNT. This DOC value (DOC_b_) was then subtracted from the DOC value measured after the single solute batch experiment with SRNOM and MWCNT (DOC_f_), in order to estimate the SRNOM adsorbed mass per mass of MWCNT, as described in Equation (1):

(1) $$q_{e}\left(SRNOM\right)=\frac{\left[DOC_{i}-\left(DOC_{f}-DOC_{b}\right)\right].V}{m_{MWCNT}}$$

where q_e_(SRNOM) is the SRNOM adsorbed mass per mass of MWCNT (mgC/g), DOC_i_ is the initial DOC concentration (mgC/L) of the SRNOM solution measured after pH and concentration adjustment and prior to the batch experiment, DOC_f_ is the DOC concentration measured after the single solute batch experiment performed in presence of SRNOM and MWCNT (mgC/L), DOC_b_ is the DOC value from the blank measured to evaluate the DOC released from MWCNT (mgC/L), V is the volume of the batch reactor (L), and m_MWCNT_ is the MWCNT mass used in each batch experiment (g).

### MS2 double layer assay and calculations

In this study, MS2 bacteriophages were used as a surrogate for enteric viruses of human health concern. MS2 (DSMZ 13767) and its associated *E. coli* host (DSMZ 5695) were purchased from DSMZ German Collection of Microorganisms and Cell Cultures (Braunsch-weig, Germany). MS2 stock solution was prepared at the beginning of the study by amplifying MS2 commercial stock solution in 1 L of *E. coli* culture. After the amplification step, the culture was filtered over a 0.22 μm PES vacuum filter membrane to remove *E. coli* cells. Cell removal prevents any proliferation of MS2 during the batch adsorption experiments. Following filtration, we additionally purified the virus stock solution using centrifugal membrane filters (Amicon Ultra-15, Merk MilliPore, Germany) before using the MS2 solution in experiments. It was previously shown that the additional centrifugal membrane filtration step is critical to remove lower molecular weight organics that may heavily interfere with MS2 adsorption to sorbent surfaces (Armanious et al., 2016a).

**Fig. 1:**
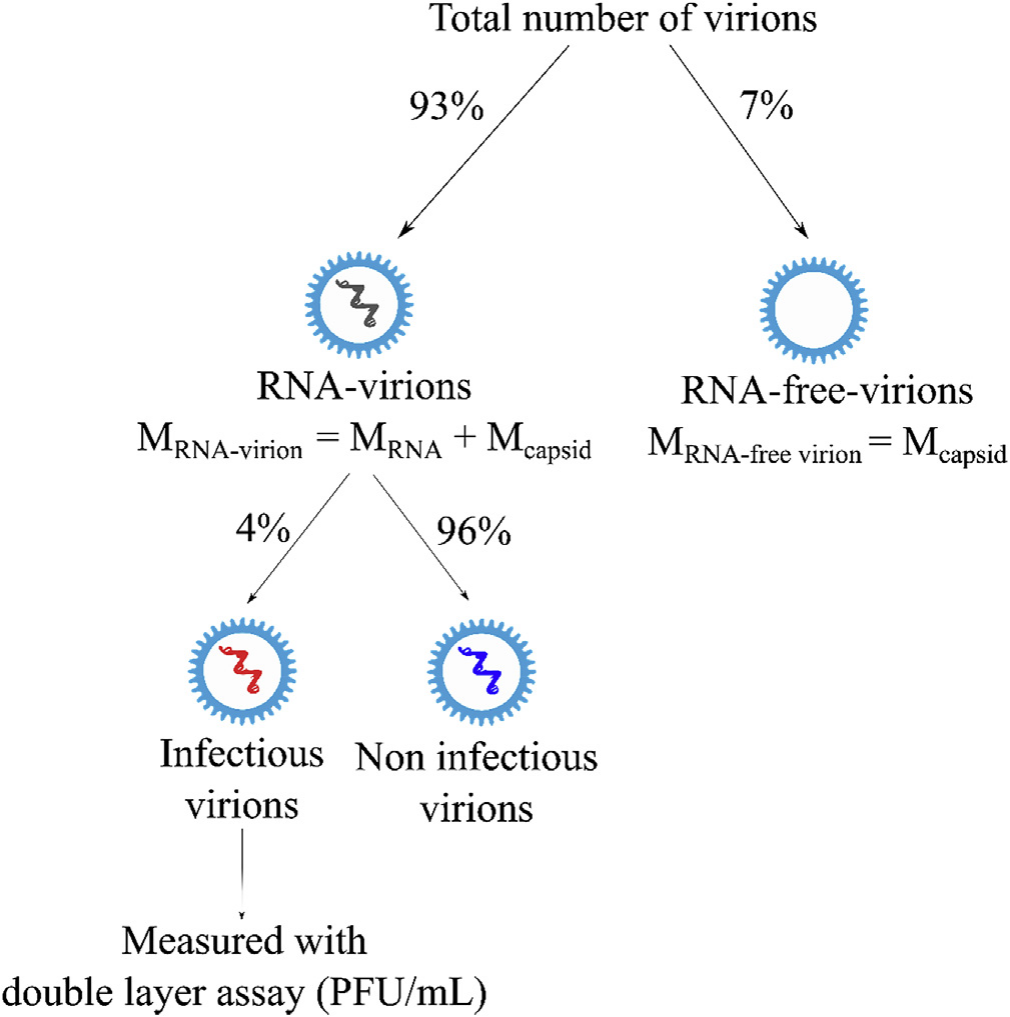
Description of the different MS2 subpopulations and their respective ratios, based on (Armanious et al., 2016a). M_RNA-virion_, M_RNA-free viron_, M_RNA_, and M_capsid_ are the masses of an individual virion containing RNA, an individual virion without RNA, the RNA, and the protein capsid, respectively. RNA-virions and RNA-free virions refer to individual virions with and without RNA in the assembled capsid.

The double agar layer assay was used to enumerate infectious bacteriophages (PFU/mL) (US EPA Method 1602, 2001; Pitol et al., 2017). Briefly, 100 μL of sample containing MS2 were mixed with 200 μL of *E. coli* host in soft agar (0.7% Agar) and poured onto a hard agar plate (1.5% Agar). After overnight incubation at 37 °C, plaques formed from the bacteriophages were counted. MS2 LRV was calculated as follows:

(2) $$LRV=log_{10}\left(\frac{C_{i}}{C_{b}}\right)$$

where C_i_ (PFU/mL) is the concentration of MS2 available for adsorption (measured from the blank experiment containing no MWCNT) and C_b_ (PFU/mL) is the final MS2 concentration after the adsorption batch experiment in the presence of MWCNT. To evaluate MWCNT MS2 adsorption capacity and compare it to the SRNOM adsorption capacity, the adsorbed mass of MS2 per mass of MWCNT (q_e_(total-virions), mg/mg) was calculated, considering that the total number of MS2 capsids in solution is higher than the total number of PFU, given that not all virions are infective (Fig. 1) (Armanious et al., 2016a).

The ratios between the different MS2 subpopulations (i.e. R_infectious-virions/RNA-virions_ = 0.04 and R_RNA-virions/total-virions_ = 0.93), combined with the PFU/mL concentration measured with the double layer assay, were used to calculate the number of RNA-virions and RNA-free-virions, referring to individual virions with and without RNA in the assembled capsid, respectively. These values were taken from the table provided in supporting information of Armanious et al. (2016a). Even if these values might be different from one study to another, due to different propagation and purification protocols, we used Armanious et al. (2016a) ratios as approximation for our work. Indeed, the MWCNT surface loading by MS2 would not be fundamentally impacted if the ratios are different.

Briefly, the number of infectious virions adsorbed onto MWCNT during batch experiment (N_infectious-virions_) was calculated using Equation (3):

(3) $$N_{infectious-virions}=R_{infectious-virions/plaque-forming-units}*V*\left(C_{i}-C_{b}\right)$$

where R_infectious-virion/plaque-forming-units_ is the ratio of infectious virions to plaque forming units (PFU), which we assumed to be equal to 1, and V as the volume used for the batch experiment (mL).

Based on N_infectious-virions_, the number of RNA-virions (N_RNA-virions_) was calculated using Equation (4):

(4) $$N_{RNA-virions}=\frac{N_{infectious-virions}}{R_{infectious-virion/RNA-virion}}$$

Then, the number of RNA-free-virions (N_RNA-free-virions_) was calculated using Equations (5), (6)):

(5) $$N_{total-virions}=\frac{N_{RNA-virions}}{R_{RNA-virions/total-virions}}$$

where N_total-virions_ is the total number of virions present in solution, accounting for both RNA-containing and RNA-free virions (Fig. 1).

(6) $$N_{RNA-free\;virions}=R_{RNA-free\;virions/total-virions}*N_{total-virions}$$

where R_RNA-free-virions/total-virions_ is the ratio of virions without RNA to total virions, assumed to be 0.07 (Armanious et al., 2016a). Based on the number of virions calculated for the subgroups and on the molar mass of a molecule of RNA (M_RNA_ = 1.83∗10^-18^ g) and a single capsid (M_capsid_ = 4.18∗10^-18^ g), the mass of virions adsorbed per mass of MWCNT was calculated using Equation (7)

(7) $$q_{e}\left(total-virions\right)=\frac{N_{RNA-virions}\left(M_{RNA}+M_{capsid}\right)+N_{RNA-free-virions}M_{capsid}}{m_{MWCNT}}$$

### Liquid chromatography coupled with organic carbon and organic nitrogen detectors (LC-OCD-OND) analysis

The NOM in the different water samples used for the co-adsorption batch experiments were characterized by size using LC-OCD-OND. This method allows to separate NOM molecules into major fractions of different sizes and chemical functions and to quantify them on the basis of organic carbon concentration. To do so, each sample was passed through a size exclusion chromatographic column from Tosoh (Toyopearl TSK HW-50S, 250 × 20 mm, with a separation range of 20 kDa-100Da). Phosphate-buffer (24 mM, pH 6.6) was used as eluent and the flow rate was set at 1 mL/min. After the chromatographic column, the flow was split into a Gräntzel thin-film reactor (C-compounds oxidation) and a special DONOX-reactor (N-compounds oxidation; not further described herein as it was not used). The upper part of the Gräntzel thin-film reactor was shielded from the UV-bulb to allow stripping of the CO_2_ present in water after mixing the sample with phosphoric acid (60 mM, pH 1.2). Then, the sample was exposed to UV-light in the lower part of the Gräntzel thin-film reactor to produce hydroxyl radical (^.^OH). C-compounds were fully oxidized by hydroxyl radicals to CO_2_, which was subsequently analyzed with a Siemens IR detector. More detailed information on LC-OCD-OND is available in (Huber et al., 2011).

The obtained chromatograms are divided in five fractions according to the NOM molecular weight (MW), i.e. biopolymers (MW ≥ 20,000 Da), humics (MW∼1000 Da), building blocks (MW∼300–500 Da), low molecular weight (LMW) organics (MW < 350 Da) and neutrals (MW < 350 Da), as described in (Huber et al., 2011).

## 3 Results and discussion

### Evaluation of the minimum MWCNT mass to comply with EPA standards for virus removal

**Fig. 2:**
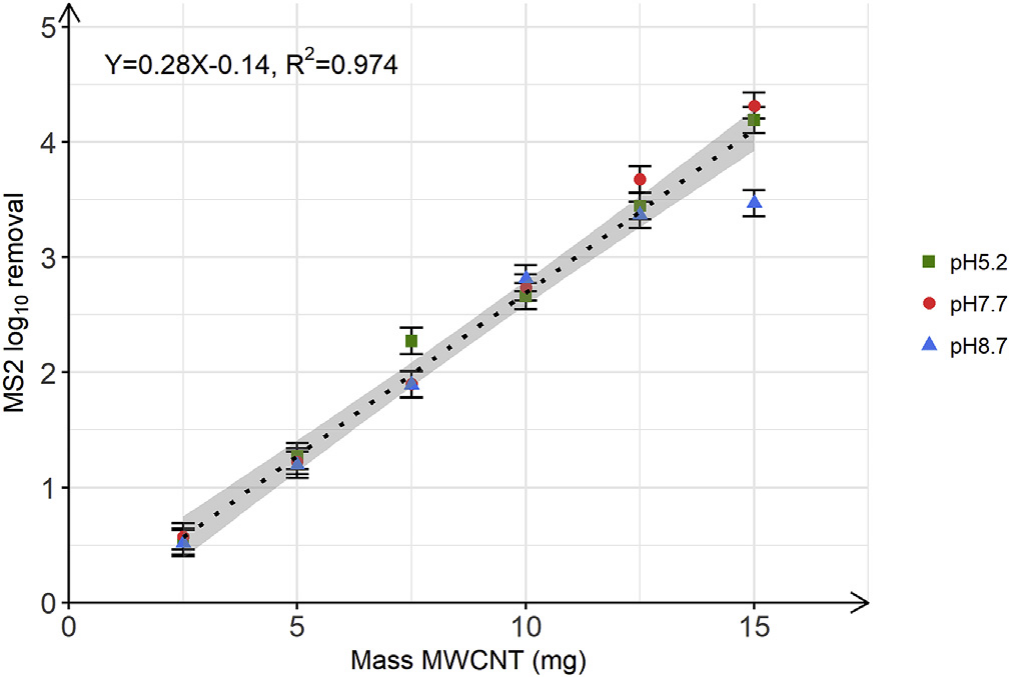
MS2 log_10_ removal as a function of MWCNT mass used during batch experiments. Green squares, red circles and blue triangles correspond to the batch experiments performed at pH 5.2, 7.7 and 8.7, respectively. The black dotted line corresponds to the single linear regression fitted to all the data at pH 5.2, 7.7 and 8.7, since pH had no significant effect on MS2 LRV. The grey zones represent the 95% confidence interval. The batch experiments were performed in duplicate at an initial MS2 concentration of 10^6^ PFU/mL. Error bars represent laboratory precision calculated using a pooled standard deviation, or weighted average of standard deviations calculated for all groups of samples, from the sets of duplicate samples. (For interpretation of the references to colour in this figure legend, the reader is referred to the Web version of this article.)

To estimate the minimum mass of MWCNT required to reach a 4 LRV and thus comply with the EPA standards for virus removal (US EPA, 2015) and to understand the pH effect on MS2 adsorption onto MWCNT, we determined MS2 LRV as a function of increasing MWCNT mass at three experimental pH of 5.2, 7.7 and 8.7 (Fig. 2). As depicted in Fig. 2, MS2 LRV was linearly correlated to the mass of MWCNT used in the batch experiments, confirming that MWCNT present MS2 adsorption sites. A multiple linear regression was calculated to evaluate both effects of MWCNT mass and pH on MS2 LRV (description of the model in section 3 of supplementary information). A significant regression equation was found (F(3,64) = 473.1, *p* < 2.2∗10^-16^), with a R^2^ of 0.955. MS2 LRV was significantly influenced by MWCNT mass (beta = 0.29, *p* < 2∗10^-16^), but neither pH 7.7 (beta = 0.04, *p* = 0.6) nor pH 8.7 (beta = 0.04, *p* = 0.6) were statistically significant relative to pH 5.2. This finding strongly suggests that the higher the MWCNT mass, the higher the MS2 LRV, as visible in Fig. 2, while pH did not significantly affect MS2 adsorption to MWCNT over the tested pH from 5.2 to 8.7. The effect of pH on adsorption mechanisms is observed if a significant change in the net surface charge occurs over the pH range tested. In our study, the negligible effect of pH on MS2 removal by MWCNT is due to the fact that both MS2 and MWCNT surfaces charge were not changed and were mainly negative over the tested pH range. Specifically, most MS2 capsid amino acids have a pKa lower than 4.5 (aspartic acid, glutamic acid) or higher than 10 (lysine) (Armanious et al., 2016a) and MWCNT are negatively charged at pH higher than 2–4.5, due to the presence of acidic groups, such as carboxylic acid moieties (pK_a_ = 4–5) (Lu and Su, 2007; Michen and Graule, 2010; Wang et al., 2011; Singh et al., 2012; Skwarek et al., 2016). Nonetheless, electrostatic repulsion is not a major limitation to MS2-MWCNT adsorption mechanisms because acidic groups have a low surface density on MWCNT (Yudianti et al., 2011; Sun et al., 2014) and they are mainly located at MWCNT tips (McClory et al., 2010; Krishnakumar et al., 2012). Furthermore, other interaction forces, such as π-π interactions, hydrogen-bounding and hydrophobic effect, can overcome electrostatic repulsion. Armanious et al. (2016a) demonstrated through QCM-D experiments the hydrophobic effect role in the adsorption of MS2 on surfaces. Based on past studies and our batch experiments results, we therefore hypothetize that MS2 primarily adsorbed through hydrophobic effect to the apolar patches present at the MWCNT surface, with MS2 having a positive hydrophathy index (Chandler, 2005; Armanious et al., 2016a).

Given that the multiple linear regression showed that pH had no significant effect on MS2 LRV, a single linear regression was calculated from all data points from the batch experiments conducted at pH 5.2, 7.7 and 8.7 to predict the MS2 LRV based on MWCNT mass (Fig. 2). A significant regression equation was found (F(1,16) = 631.1, *p* = 2.8∗10^-14^), with a R^2^ of 0.974. MS2 LRV predicted is equal to -0.14 + 0.28 (MWCNT mass) when MWCNT mass is measured in milligrams. At an initial MS2 concentration of 10^6^ PFU/mL, MS2 LRV increased by 0.28 for each mg of MWCNT, over the pH range tested. Fig. 2 shows that a minimum mass of 15 mg MWCNT was required to reach the 4 LRV of MS2, as imposed by EPA regulations (US EPA, 2015). Notably, because MS2 LRV was linearly correlated with MWCNT mass in the pH range tested, higher levels of virus LRV are expected by increasing mass of MWCNT. These results are in accordance with other studies that showed higher virus removal efficiency with increasing CNT mass onto filters (Brady-Estévez et al., 2010b; Park and Hwang, 2014). We note that Brady-Estévez et al. (2010c) used a lower amount of MWCNT of 3 mg per filter at a water chemistry (10 mM NaCl and pH 5.5) that was similar to what we used here, but obtained 5.38 ± 0.80 LRV for MS2. This higher removal in that study, as compared to our values, likely resulted from the use of a different MWCNT material in Brady-Estévez et al. (2010c) (NanoTechLabs, Inc., 17 ± 9 nm diameter, 91 ± 21 μm length). MWCNT properties vary between suppliers and even batches (Poulsen et al., 2015, 2016). Glomstad et al. (2016) showed that CNT adsorption capacity of phenanthrene was distinct between two types of MWCNT, while Brady-Estévez et al. (2010c) reported a higher MS2 removal with a MWCNT filter than with a SWCNT filter. Another factor that can explain the difference between our results and those of Brady-Estévez et al. (2010c) is that the filtration experiments conducted by Brady-Estévez et al. (2010c) combined MS2 physical retention by the entangled MWCNT layer with adsorption on MWCNT, while we only assessed MS2 adsorption to MWCNT in our experimental setup. However, in regards to the drastic MS2 LRV decrease observed by Brady-Estévez et al. (2010c) during the filtration of virus in co-solute systems, it can be hypothesized that adsorption was the main pathway by which virus was removed from water by MWCNT filters. MS2 adsorption onto MWCNT is expected to be largely driven by the hydrophobic effect, as mentioned above.

### Concentration dependent adsorption of MS2 by MWCNT

**Fig. 3:**
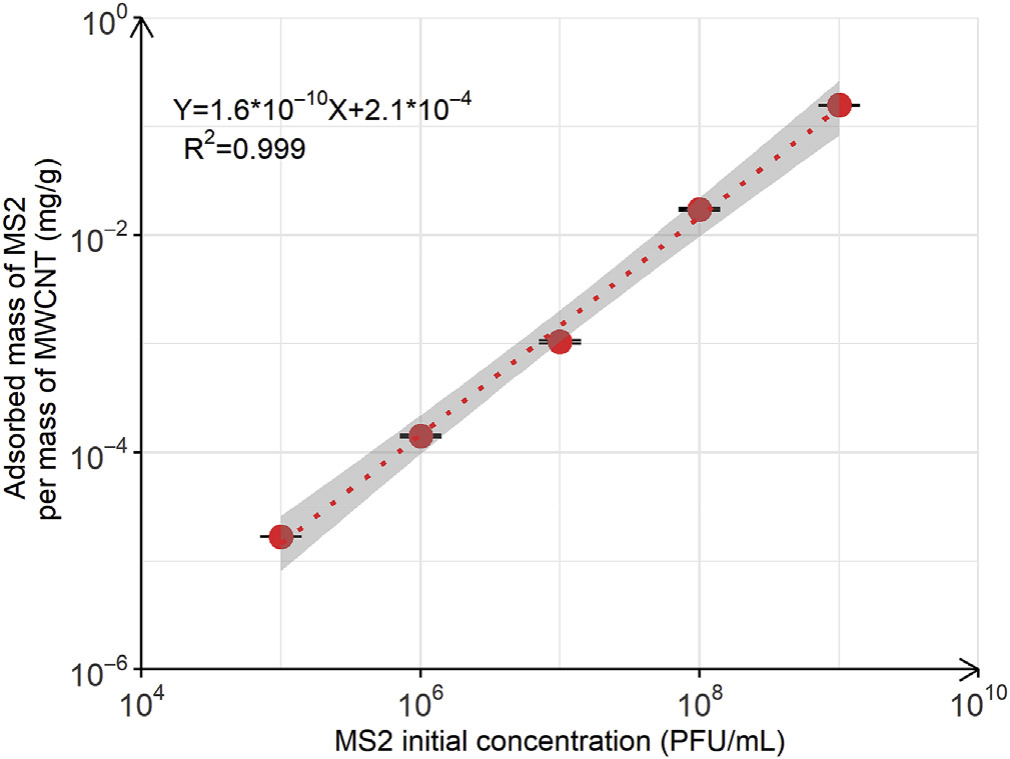
MS2 adsorbed mass per mass of MWCNT as a function of MS2 initial concentration ranging from 10^5^ to 10^9^ PFU/mL. The batch experiments were performed in duplicate at pH 7.7 with a fixed MWCNT mass of 15 mg. The red dotted line corresponds to the single linear regression. The grey zone corresponds to the 95% confidence interval of the linear regression fit. Error bars represent laboratory precision calculated using a pooled standard deviation, or weighted average of standard deviations calculated for all groups of samples, from the sets of duplicate samples. (For interpretation of the references to colour in this figure legend, the reader is referred to the Web version of this article.)

The effect of initial MS2 concentration on MS2 adsorption by MWCNT was studied to evaluate if MWCNT saturation by MS2 could be reached at a mass of 15 mg MWCNT. Information on the maximum adsorbed mass or number of MS2 capsids per mass of MWCNT would allow estimating the volume of virus-free filtered water that can be obtained from one MWCNT filter, assuming a human virus concentration of 10^3^ PFU/L or 10^1^-10^3^ gene copy/L in river water and that these viruses adsorb similarly to MS2 (Haramoto et al., 2010; Hamza et al., 2011; Goh et al., 2019). The adsorbed mass of MS2 per mass of MWCNT was therefore plotted as a function of MS2 initial concentration, as shown in Fig. 3.

A single linear regression was calculated to predict the adsorbed mass of MS2 per mass of MWCNT based on the initial MS2 concentration (Fig. 3). A significant regression equation was found (F(1,3) = 2.5∗10^4^, *p* = 5.6∗10^-7^), with a R^2^ of 0.999. The adsorbed mass of MS2 per mass of MWCNT predicted is equal to 2.1∗10^-4^+1.6∗10^-10^ (MS2 initial concentration) when MS2 initial concentration is measured in PFU/mL. The adsorbed mass of MS2 per mass of MWCNT increased by 1.6∗10^-10^ mg/g per PFU/mL of MS2. At the maximum initial MS2 concentration tested (i.e. 10^9^ PFU/mL), the adsorbed mass of MS2 per mass of MWCNT was 0.16 ± 0.002 mg/g, corresponding to a total capsid number of 4∗10^11^ ± 4∗10^9^ adsorbed per 15 mg (corresponding to a MWCNT surface area of 1.75∗10^4^ cm^2^).

Based on the MS2 capsid diameter of approximately 28.8 nm, we estimated that at an initial MS2 concentration of 10^9^ PFU/mL, the total number of virions adsorbed to the MWCNT covered a surface of 2.6 cm^2^, corresponding to only 0.015% of the MWCNT total surface. This is approximately 2000 times lower than the hypothetical maximum surface coverage (i.e., 24–40% of surface covered by MS2) if one assumes random sequential adsorption of individual MS2 virions up to the surface jamming limit, following Armanious et al. (2016a). We note that in the same study, adsorption of MS2 leveled off at a concentration of approximately 1800 ng/cm^2^, while in our study the highest adsorbed MS2 concentration was only 0.13 ng/cm^2^ (at a MS2 concentration of 10^9^ PFU/mL). The differences in the adsorbed mass solely reflect the different experimental set-ups for adsorption in both studies. In Armanious et al. (2016a), MS2 was continuously delivered at a constant virus inflow concentration over a QCM-D sensor, until the jamming limit of MS2 on the sensor surface was reached. In our study, the initial MS2 concentration was significantly smaller than the concentration that would have been needed to reach the jamming limit (i.e. 10^12^ PFU/mL, calculated from Armanious et al. (2016a) maximum surface coverage and MWCNT surface area available for adsorption). Furthermore, reduction in surface area available for MS2 adsorption due to MWCNT aggregation and/or bundle formation was not accounted for in our calculation. It is therefore difficult to predict the maximum amount or mass of virus that can be adsorbed per mass of MWCNT, but Fig. 3 shows that even with an initial concentration of 10^9^ PFU/mL, the jamming limit of MWCNT surface was not reached.

### Concentration dependent adsorption of SRNOM to MWCNT

**Fig. 4:**
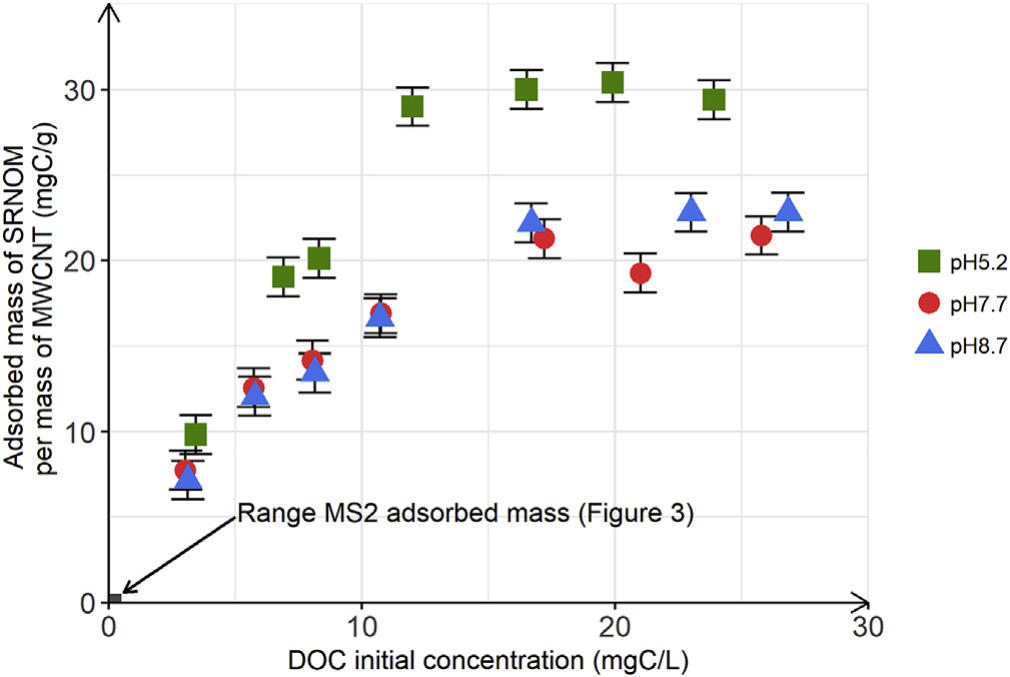
SRNOM adsorbed mass per mass of MWCNT as a function of DOC initial concentration of SRNOM used as a coadsorbate. The batch experiments were performed in duplicate with a fixed MWCNT of 15 mg. Green squares, red circles and blue triangles correspond to the batch experiments performed at pH 5.2, 7.7 and 8.7, respectively. The red rectangle at the left bottom corresponds to the range of MS2 adsorbed mass as a function of MS2 initial concentration (Fig. 3). Error bars represent laboratory precision calculated using a pooled standard deviation, or weighted average of standard deviations calculated for all groups of samples, from the sets of duplicate samples. (For interpretation of the references to colour in this figure legend, the reader is referred to the Web version of this article.)

Natural waters containing viruses also contain NOM as a co-solute that may interfere with virus removal by MWCNT-filters, as already observed by Brady-Estévez et al. (2010c). It is therefore critical to assess the effect of initial NOM concentration on NOM adsorption onto MWCNT, to evaluate the potential NOM competitive co-adsorption effect with viruses. To do so, we conducted single solute NOM adsorption experiments onto MWCNT at varying initial NOM concentrations. The resulting adsorbed amounts of NOM per mass of MWCNT are plotted as a function of the initial DOC concentration in Fig. 4.

Fig. 4 shows that for low initial DOC concentrations the adsorbed mass of SRNOM per mass of MWCNT increased as the initial DOC concentration increased. For higher initial DOC concentrations, contrary to what was observed with MS2 (Fig. 3), the adsorbed mass of SRNOM per mass of MWCNT attained a threshold, suggesting that surface saturation of MWCNT was reached. The maximum adsorbed mass of SRNOM per mass of MWCNT was 20.7 ± 1.22 mgC/g and 22.6 ± 0.37 (i.e. an adsorbed mass per surface of MWCNT equals to 17.7 ngC/cm^2^ and 19.3 ngC/cm^2^, respectively) at pH 7.7 and 8.7, respectively. These values are in agreement with Jeong et al. (2017) who reported a saturation concentration around 20 mgC/g after batch experiments with MWCNT and SRNOM. π-π interactions, electrostatic interactions, hydrogen-bonding and the hydrophobic effect are the main driving forces SRNOM-MWCNT interactions (Ateia et al., 2017). In contrast to MS2, the adsorption of SRNOM to MWCNT was pH dependent: the maximum adsorbed mass of SRNOM per mass of MWCNT at pH 5.2 was 29.9 ± 0.5 mgC/g and higher than the value measured at pH 7.7 and 8.7. The higher adsorption of SRNOM to MWCNT at the lower tested pH likely resulted from SRNOM being less negatively charged at this pH, and thus possibly adopting a more compact conformation and consequently denser packing on the MWCNT surface as compared to the higher pH. Adoption of a more compact conformation of HA assemblies in solution at lower pH was previously demonstrated for Suwannee river humic acid (SRHA) by photon correlation spectroscopy (PCS) (i.e., the hydrodynamic diameter of the assemblies decreased from approximately 60 nm–10 nm when decreasing the pH from 7.5 to 4.5 at constant IS = 10 mM (Baalousha et al., 2006)). The more compact conformation and denser packing can be explained by weaker intra- and inter-molecular electrostatic repulsion in and between SRNOM assemblies on the sorbent surface, as previously reported in Armanious et al. (2014) and de Melo et al. (2016). Higher adsorption of NOM to CNT at lower pH was also observed by Engel and Chefetz (2016) who reported that the saturation value of dissolved organic matter from composted biosolids on SWCNT was approximately 1.8 times higher at pH 4 than at pH 7 and pH 10.

To calculate the surface coverage of MWCNT by SRNOM we assumed that the representative formula of a NOM assembly is C_9_H_9_NO_6_ (i.e. 1.79∗10^-19^ mgC), that assemblies can be modeled as rigid spheres having a diameter of 1 nm (Armanious et al., 2016b) and that all MWCNT surface area is available for adsorption. At pH 7.7, we therefore calculated that 78% of the MWCNT surface was covered by SRNOM, when the maximum adsorbed mass of SRNOM per mass of MWCNT was reached (i.e. 20.7 ± 1.22 mgC/g) (Fig. 4). Yet, MWCNT tend to form bundles and aggregates, 78% of surface coverage might correspond to a saturation of all MWCNT adsorption sites available. The MWCNT surface coverage value obtained for SRNOM is 5∗10^6^ times higher than the surface coverage of MS2 on MWCNT that we calculated for the experiment conducted at the highest tested initial MS2 concentration (i.e. 10^9^ PFU/mL). Even for initial DOC concentrations where MWCNT surface saturation by SRNOM was not reached, MWCNT surface coverage by SRNOM was significantly higher than the surface coverage observed for MS2, irrespectively the initial MS2 concentration. Indeed, at low SRNOM concentration (i.e. 2.5 mgC/L), the calculated MWCNT surface coverage by adsorbed SRNOM was approximately 29%. This drastic difference between SRNOM and MS2 MWCNT surface coverage might be explained by the comparable or higher affinity of SRNOM to MWCNT in comparison to MS2 and/or to higher concentrations of SRNOM assemblies than MS2 capsids at the tested concentrations (Fig. 4). Our experimental findings suggest that NOM is expected to have a major effect on MS2 adsorption onto MWCNT, even at low NOM concentration.

### Competitive co-adsorption of MS2 and SRNOM to MWCNT

**Fig. 5:**
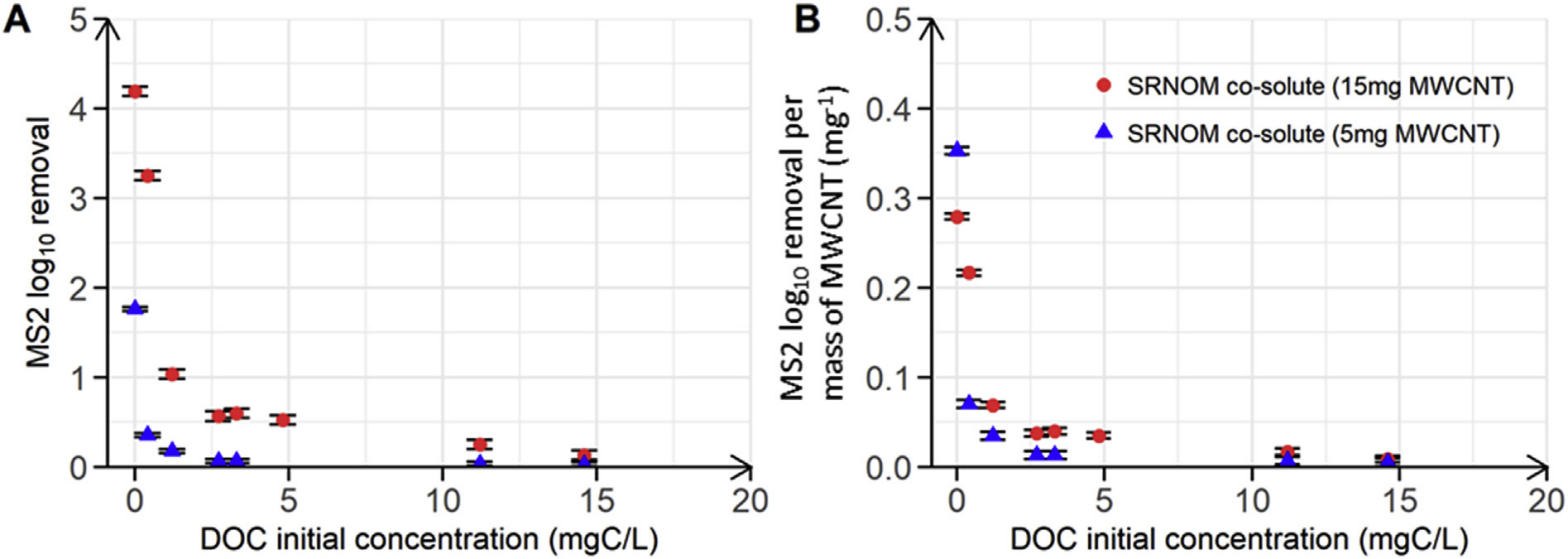
(A) MS2 log_10_ removal as a function of DOC initial concentration of SRNOM used as a co-solute and (B) MS2 log_10_ removal normalized to the MWCNT mass used in the experiment as a function of DOC initial concentration of co-solute SRNOM. The red circles and the blue triangles correspond to batch experiments performed in duplicate with MWCNT masses of 15 mg and 5 mg, respectively. Both batch experiments were performed at pH 7.7 and at an initial MS2 concentration of 10^6^ PFU/mL. Error bars represent laboratory precision calculated using a pooled standard deviation, or weighted average of standard deviations calculated for all groups of samples, from the sets of duplicate samples. (For interpretation of the references to colour in this figure legend, the reader is referred to the Web version of this article.)

To evaluate competitive co-adsorption of NOM and MS2 on MWCNT, both at low and high NOM concentrations, we performed batch co-adsorption experiments with 5 mg and 15 mg of MWCNT and at different initial SRNOM concentrations. For both MWCNT masses, the presence of SRNOM resulted in significant decreases in MS2 adsorption and hence MS2 LRV, even at the lowest SRNOM concentrations tested (Fig. 5A). SRNOM presence therefore decreases MS2 access to MWCNT adsorption sites possibly due to the competition for the same adsorption site, since SRNOM adsorption to MWCNT is also driven by hydrophobic effect (Ateia et al., 2017). In addition, SRNOM might adsorb to other adsorption sites and consequently block the access to MS2 to MWCNT adsorption sites. At the low initial SRNOM concentration of 0.4 mgC/L, the MS2 LRV decreased by 22% (from 4.2 to 3.3 LRV) and by 80% (from 1.8 to 0.4 LRV) for batch reactors containing 15 and 5 mg of MWCNT, respectively. These experimental results show that the initial DOC concentration has a major effect on the competitive adsorption of MS2 and SRNOM onto MWCNT. Indeed, at an initial SRNOM concentration of 0.4 mgC/L in the batch reactors (20 mL), the estimated number of SRNOM assemblies was 10^8^ times higher than the number of capsids (3∗10^7^ capsids in 20 mL). The finding of competitive co-adsorption between MS2 and SRNOM at such low SRNOM concentrations is in apparent contrast to the finding in Brady-Estévez et al. (2010c) who showed that MS2 LRV was not affected by SRNOM at a concentration of 1 mgC/L. Yet, as suggested in the introduction, this observation could also be due to the a short contact time of the SRNOM with the filter surfaces during filtration, in comparison with our batch experiments, resulting in a lower competitive effect at low NOM concentrations. Furthermore, our findings are in good agreement with reported competitive co-adsorption of MS2 and SRHA onto self-assembled monolayers of alkyl-thiols formed from ethanolic solution of cysteamine (SAM-NH_3_^+^) surface as investigated using QCM-D (Armanious et al., 2016b). Specifically, the adsorbed mass of MS2 on the SAM-NH_3_^+^ surface decreased from 2300 ng/cm^2^ in the absence of SRHA to 1400 and 400 ng/cm^2^ when solutions contained SRHA as co-solute at concentrations of 0.25 mgC/L and 2.5 mgC/L, respectively. The low SRHA concentration of 0.25 mgC/L therefore decreased MS2 adsorbed masses by 39% relative to the adsorbed mass in the absence of SRHA. This decrease is comparable to the decrease shown in Fig. 5A in presence of SRNOM. Our results thus highlight that the presence of NOM as co-solute decreases the capability of MWCNT to adsorb viruses, irrespective of the initial NOM concentration.

MS2 LRV decreased exponentially with increasing initial SRNOM concentration until reaching a LRV close to zero for both MWCNT masses at the highest tested SRNOM concentrations (Fig. 5A). MS2 LRV exponential decrease was more pronounced at lower MWCNT masses: MS2 LRV decreased to values close to zero for initial SRNOM concentration at 2.5 mgC/L and above when using 5 mg MWCNTs, whereas similar LRV values were reached only above 10 mgC/L SRNOM when using 15 mg MWCNT (Fig. 5A). These results confirmed that the higher the MWCNT surface area, the more adsorption sites and therefore the less competition between SRNOM and MS2 occurred, as already suggested by the linear increase of MS2 LRV with increasing MWCNT mass in Fig. 2. The decrease in competition with increasing MWCNT is also supported by Fig. 5B in which we replotted the data from Fig. 5A, but normalized MS2 LRV to the mass of MWCNT used. This normalization resulted in similar decrease in MS2 LRV for experiments with 5 mg and 15 mg MWCNT. The competitive co-adsorption effect at a given initial virus concentration and SRNOM concentration increased as the number of adsorption sites on MWCNT decreased, as a result of decreasing MWCNT mass.

In the application of MWCNT-filters designed for water treatment, water and thus NOM will be continuously delivered to the MWCNT surface. Furthermore, the concentration of NOM is expected to be much higher than the concentration of viruses. The NOM will therefore adsorb to the MWCNT surface until the entire surface is saturated, irrespectively of which MWCNT mass is used to prepare a MWCNT-filter and the initial NOM concentration in the natural water.

**Fig. 6:**
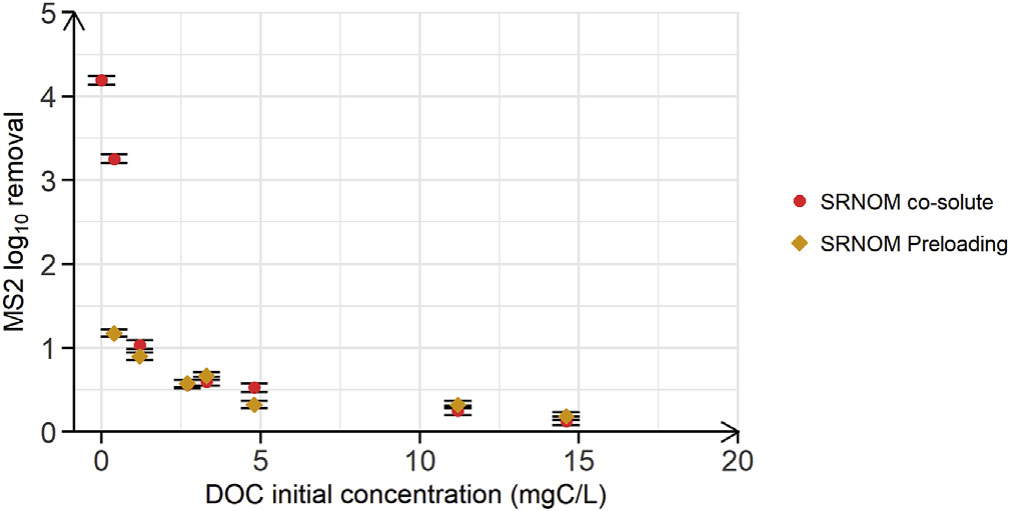
MS2 log_10_ removal by MWCNT as a function of DOC initial concentration of SRNOM. The red circles correspond to the batch experiment performed with SRNOM as co-solute and a mass of MWCNT of 15 mg, and is re-plotted from Fig. 5A. These results are used as reference to the batch experiments in which we pre-adsorbed SRNOM to the MWCNT for 3 h (in the absence of MS2), followed by addition of MS2 and a second period of equilibration for 3 h (orange diamonds). Both batch experiments were performed in duplicate at pH 7.7, at a MWCNT mass of 15 mg and at an initial MS2 concentration of 10^6^ PFU/mL. Error bars represent laboratory precision calculated using a pooled standard deviation, or weighted average of standard deviations calculated for all groups of samples, from the sets of duplicate samples. (For interpretation of the references to colour in this figure legend, the reader is referred to the Web version of this article.)

Because the SRNOM adlayer on the MWCNT is negatively charged, negatively charged viruses (like many enteric viruses) would therefore be electrostatically repelled and not retained by the filter. Electrostatic repulsion of viruses from NOM adlayers was already demonstrated by Armanious et al. (2016b): MS2 did not adsorb onto QCM-D sensors that were pre-coated by SRHA. In order to verify that the SRNOM adlayer on MWCNT indeed impaired MS2 adsorption (and hence lowered MS2 LRV), we first preloaded MWCNT with SRNOM at different initial concentrations (3 h equilibration) before adding MS2 to the batch reactors (Fig. 6). As compared to the experiments in which the two co-solutes were added simultaneously, the pre-addition of SRNOM and hence the pre-formation of a SRNOM adlayer on MWCNT surfaces resulted in pronounced decreases in MS2 LRV at low SRNOM initial concentrations. At an initial SRNOM concentration of 0.4 mgC/L, MS2 LRV decreased from 4.2 LRV to 3.3 LRV, when MS2 and SRNOM were simultaneously added, to only 1.2 LRV, when added sequentially. The LRV of MS2 was therefore about 3 times lower than the value that we observed for the batch experiment in which SRNOM and MS2 were simultaneously added to 15 mg of MWCNT. These results confirm that the formation of a negatively charged NOM adlayer on the MWCNT surface impaired adsorption of MS2. At higher initial NOM concentrations (>1 mgC/L), the MS2 LRV was comparable, irrespectively of whether SRNOM was preloaded to the MWCNT or simultaneously added with MS2. These SRNOM concentrations therefore were sufficiently high for SRNOM to outcompete MS2 for adsorption sites on the MWCNT, even when the two adsorbates were simultaneously added (Figs. 5A and 6).

Our results strongly suggest that virus adsorption by MWCNT-based filters is impractical from waters that also contain NOM. To demonstrate that the results obtained with SRNOM are transferrable to natural waters with different types of NOM, we subsequently analyzed competitive co-adsorption of MS2 and NOM in different natural water samples. Furthermore, we assessed the effect of Ca^2+^ on MS2 removal.

### Effect of water calcium concentration and water source on the competitive co-adsorption of MS2 and NOM to MWCNT

**Fig. 7:**
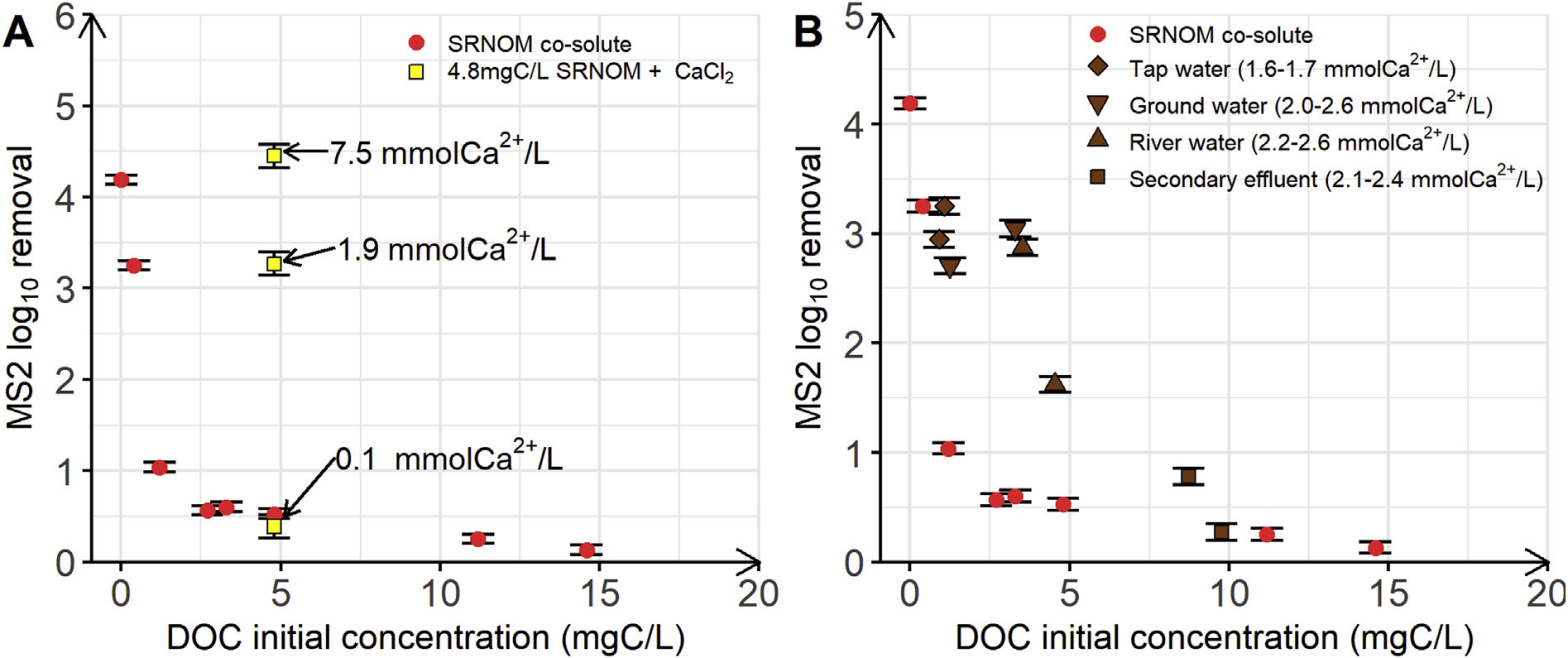
MS2 log_10_ removal by MWCNT as a function of DOC initial concentration. A) Assessment of the effect of dissolved Ca^2+^ concentration on MS2 LRV at a constant NOM concentration of 4.8 mgC/L. Yellow squares correspond to the batch experiments in the presence of added Ca^2+^ to concentrations of 0.1 mmol/L, 1.9 mmol/L and 7.5 mmol/L. (B) Assessing the effect of different NOM types and concentrations in natural water samples on MS2 LRV. The brown diamonds, inverse brown triangles, brown triangles and brown squares correspond to the batch experiments performed with tap water, ground water, river water and secondary effluent, respectively. In the case of natural waters, we did not adjust the pH. It was equal to 7.4, 7.5, 7.9–8.1 and 8.0–8.3 for tap water, ground water, river water and secondary effluent, respectively. The red circles in panels (A) and (B) are data replotted from Fig. 5A for comparison (pH 7.7 with varying initial SRNOM concentrations, a MWCNT mass of 15 mg, and an initial MS2 concentration of 10^6^ PFU/mL). All batch experiments were performed with a MWCNT mass equal to 15 mg and a MS2 concentration of 10^6^ PFU/mL. Error bars represent laboratory precision calculated using a pooled standard deviation, or weighted average of standard deviations calculated for all groups of samples, from the sets of duplicate samples. (For interpretation of the references to colour in this figure legend, the reader is referred to the Web version of this article.)

Adsorption of MS2 to MWCNT (and other negatively charged viruses) from natural waters will likely be influenced by factors beyond the concentration of NOM as a co-solute. For example, Brady-Estévez et al. (2010c) showed that adding Ca^2+^ (1 mM CaCl_2_) to a solution containing MS2 and alginate increased by 1.2 the MS2 LRV by a MWCNT-filter. The authors ascribed this increased removal in the presence of Ca^2+^ to the formation of cation bridges between negatively charged groups in the NOM adlayer and on the MS2 surface. Evidence for such cation bridges was provided already in earlier studies (Jermann et al., 2007; Pham et al., 2009). Enhanced virus LRV in presence of dissolved Ca^2+^ could imply that MWCNT may be used as a filter material for virus removal for waters that contain high Ca^2+^ concentrations or if Ca^2+^ is added to waters prior to the filtration step. To assess whether Ca^2+^ alleviates competition by allowing MS2 to adsorb to NOM adlayer, we performed batch experiments with a fixed SRNOM concentration (i.e. 4.8 mgC/L) and three different CaCl_2_ concentrations (i.e., 0.1 mmol/L, 1.9 mmol/L and 7.5 mmol/L). LRV of MS2 increased with increasing Ca^2+^ concentration (Fig. 7A). Specifically, at a concentration of 0.1 mmolCa^2+^/L, MS2 LRV was equal to 0.4 ± 0.3, but it increased to 3.3 ± 0.3 and 4.5 ± 0.3 at 1.9 mmol/L and 7.5 mmol/L respectively (Fig. 7A, yellow squares). This finding implies that Ca^2+^ enhanced MS2 adsorption onto SRNOM adlayer, likely by forming cationic bridges, based on previous observation (Jermann et al., 2007; Pham et al., 2009). However, direct evidence confirming cationic bridges between NOM-adlayer and MS2 at the surface of the MWCNT remains missing.

**Fig. 8:**
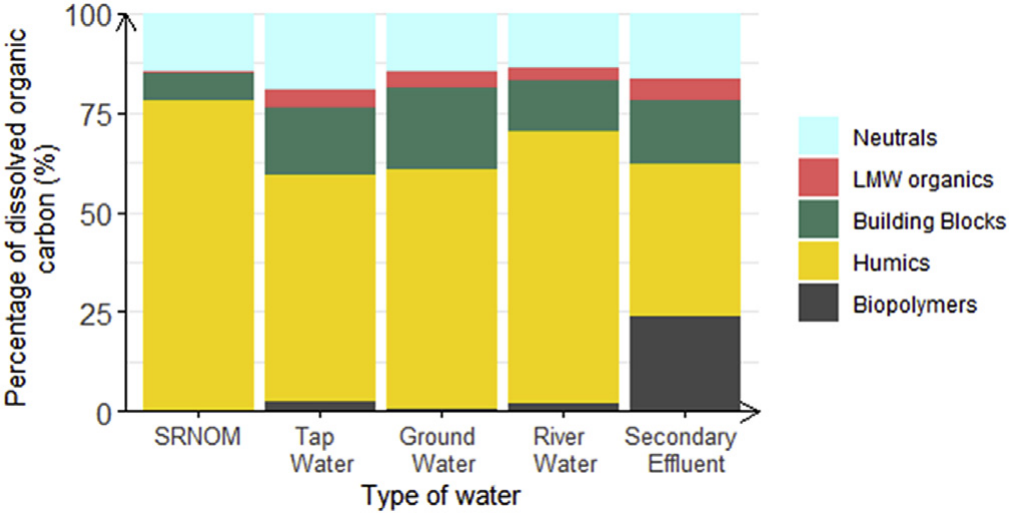
Chemical composition of NOM samples (tap water, ground water, river water and secondary effluent) determined by LC-OCD-OND. The fractions included neutrals (light blue), LMW organics (red), building blocks (green), humics (yellow) and biopolymers (dark grey) (all expressed in % of total dissolved organic carbon) fraction measured in the natural water samples used for the batch experiments. Natural water sampled in December 2018. (For interpretation of the references to colour in this figure legend, the reader is referred to the Web version of this article.)

Given that Ca^2+^ alleviated competitive co-adsorption of SRNOM and MS2, we also conducted additional MS2 removal experiments with natural waters containing different types of NOM and a different ionic matrix than the dilution buffer used for the co-solutes batch experiments, to compare them to the previous batch experiments. Tap, ground and river water, as well as wastewater treatment plant secondary effluent, were sampled twice (December 2018 and January 2019) to obtain samples that contain a range of NOM types and sources, as well as Ca^2+^ concentration. The water sources ranged in Ca^2+^ concentrations from 1.6 to 2.6 mmolCa^2+^/L, and in DOC concentrations between 0.91 and 9.77 mgC/L. We characterized differences in size and chemical functions of the NOM in the different samples by LC-OCD-OND (Fig. 8). Specifically, the percentage of the dissolved organic carbon that was present as biopolymers, humics, building blocks, LMW organics and neutrals ranged from 0.2 to 24%, 37.9 to 78%, 6.7 to 20.4%, 0.5 to 5.4% and 13.6 to 19.5%, respectively (Fig. 8). For DOC initial concentrations between 0.91 and 3.53 mgC/L, MS2 LRV varied between 2.7 and 3.3, which was higher than MS2 LRV observed for SRNOM co-solute batch experiments at similar initial DOC concentrations conducted without Ca ^2+^ (Fig. 7B, red circles). Interestingly, these LRV values were comparable for the different natural NOM samples, irrespectively the initial DOC concentration (in the range 0.91–3.53 mgC/L) or the chemical composition in NOM (Fig. 8), contrary to what was measured for SRNOM co-solute experiment without Ca^2+^. The difference in LRV value and trend observed between the batch experiments conducted in natural waters, in the DOC range of 0.91 and 3.53, and the SRNOM co-solute experiments conducted without Ca^2+^ may be due to the presence of divalent and trivalent cations in natural waters that might mitigate the competitive co-adsorption of MS2 and NOM on MWCNT, like Ca^2+^ that was present at similar concentrations in all natural water samples. Indeed, the MS2 LRV measured for natural waters having an initial DOC concentration between 0.91 and 3.53 mgC/L was in a similar range than the MS2 LRV measured for the batch experiment performed with an initial SRNOM concentration of 4.8 mgC/L and a Ca^2+^ concentration of 1.9 mmolCa^2+^/L.

Overall, the natural NOM samples, similar to SRNOM, lowered MS2 LRV in comparison with single solute experiment with MS2 and MWCNT, indicative of competitive co-adsorption. At low initial DOC concentrations, the competition was likely alleviated by the presence of divalent or trivalent cations, especially Ca^2+^, as described above (Fig. 7). However, for initial DOC concentrations higher than 3.53 mgC/L, MS2 LRV also decreased as initial NOM concentration increased in the NOM samples, until reaching values close to zero for the batch experiments performed with secondary effluent, irrespective Ca^2+^ concentration. The findings highlight that competitive co-adsorption is independent of natural water types and that the competition increased with increasing NOM:virus ratios and decreasing Ca^2+^ concentrations (Figs. 7 and 8).

We therefore conclude that while MWCNT may be a promising material for adsorption of virus in the absence of NOM, its application to virus removal in natural waters is expected to not be suitable due to the presence of NOM, which undergoes competitive co-adsorption with the viruses. Competitive suppression of virus removal by NOM is expected to occur for NOM that vary broadly in chemical composition and also in waters that show varying Ca^2+^ concentrations.

## 4 Conclusion

• This study provides evidence for competitive co-adsorption of MS2 and NOM, thereby highlighting that the occurrence of NOM as a co-solute in natural waters questions the suitability of MWCNT as a virus adsorbent to produce filters for POU systems. Despite the fact that MWCNT show outstanding adsorption properties in controlled systems, we show that the presence of NOM, even at low DOC concentration and in presence of Ca^2+^, competitively suppresses virus adsorption.
• Adsorption of SRNOM onto MWCNT impairs MS2 adsorption due to the formation of a negatively charged SRNOM adlayer on the MWCNT surface, resulting in electrostatic repulsion of the net negatively charged MS2 from the adlayer. This effect occurs irrespective of which MWCNT mass is used and irrespective of the initial NOM concentration. Indeed, batch experiments with MS2 and SRNOM as co-solutes showed that MS2 LRV decreased exponentially with increasing SRNOM concentration down to very low LRV values at the highest tested SRNOM concentrations.
• Batch adsorption experiments with dissolved Ca^2+^ and natural water samples showed that NOM of different sources and with varying chemical compositions competed with MS2 for adsorption onto the MWCNT. Although dissolved Ca^2+^ presence might punctually alleviated the NOM competitive effect, we expect that for most natural waters, where virus:NOM ratio is low, MWCNT should not be considered promising adsorbents for the removal of negatively charged human viruses.

## Declaration of competing interest

The authors declare that they have no known competing financial interests or personal relationships that could have appeared to influence the work reported in this paper.
