# Supplementary material for: Competitive co-adsorption of bacteriophage MS2 and natural organic matter onto multiwalled carbon nanotubes
Source: Water Res X. 2020 Jun 10;9:100058. doi: 10.1016/j.wroa.2020.100058 (PMC7322093; doi:10.1016/j.wroa.2020.100058)
Supplement: Multimedia component 1 [file mmc1.pdf]

Competitive co-adsorption of bacteriophage MS2 and Natural Organic Matter onto Multiwalled Carbon Nanotubes

Céline Jacquin[1], Diya Yu[1], Michael Sander[2], Kamila Domagala[3,4], Jacqueline Traber[1], Eberhard Morgenroth[1,5], Timothy R. Julian[1,6,7]

[1] Eawag, Swiss Federal Institute of Aquatic Science and Technology, 8600 Dübendorf, Switzerland.

[2] Institute of Biogeochemistry and Pollutant Dynamics (IBP), Department of Environmental Systems Science, ETH Zürich, Zürich 8092, Switzerland

[3] Empa, Swiss Federal Laboratories for Materials Science and Technology, Laboratory for High Performance Ceramics, Überlandstrasse 129, 8600 Dübendorf, Switzerland

[4] AGH, University of Science and Technology, Faculty of Materials Science and Ceramics, al. Mickiewicza 30, 30-059 Krakow, Poland.

[5] ETH Zürich, Institute of Environmental Engineering, 8093 Zürich, Switzerland

[6] Swiss Tropical and Public Health, P.O. Box, 4001 Basel, Switzerland.

[7] University of Basel, P.O. Box, 4002 Basel, Switzerland

Supplementary information

1. MWCNT purity

Since MWCNT contain metallic impurities, which proportions and composition vary from one supplier to another, we reported bellow the mass of impurities per mass of MWCNT of the MWCNT used in this study (*CheapTubes, LOT number 180320; USA)*.

Table S1: ICP-MS measurements of MWCNT digestates to quantify the metallic impurities (i.e. manganese, iron, strontium, barium and nickel). Digestates resulted from the digestion of a fixed mass of MWCNT into Teflon vials containing 4 mL HNO_3 (65%) and 0.5 mL H_2O_2. Measurements were performed with samples (n=6) taken at different spots of the same MWCNT batch used in this study (LOT number 180320). The values reported in the table stand for the average values measured for the 6 samples and their corresponding standard deviations.

Mn	Fe	Sr	Ba	Ni
mg/kg	mg/kg	mg/kg	mg/kg	mg/kg

0.05 ±0.01	0.44 ±0.43	0.20 ±0.04	0.51 ±0.04	115.19 ± 8.14

2. Preliminary tests to evaluate the batch experiment time required to reach equilibrium

Before starting the batch experiments, we first evaluated the time required to reach adsorption equilibrium of both SRNOM (Figure S1) and MS2 (Figure S2) onto the MWCNT used in this study.

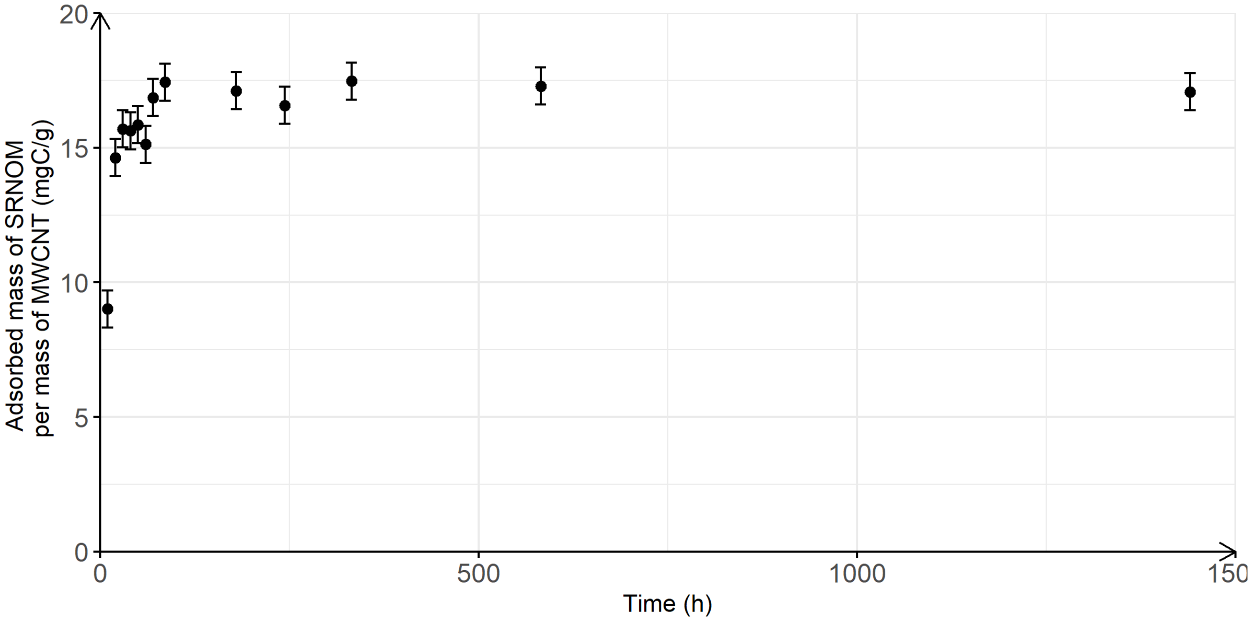

Figure S 1: SRNOM adsorbed mass per mass of MWCNT as a function of time. This batch experiment was performed at pH 7.7, with a mass of MWCNT of 15 mg and SRNOM solution at an initial concentration of 10 mgC/L. This experiment served to evaluate the time required for the batch experiments to attain apparent NOM adsorption equilibrium. One reactor was sampled in duplicate at different times. Error bars represent laboratory precision calculated using a pooled standard deviation, or weighted average of standard deviations calculated for all groups of samples, from the sets of duplicate samples.

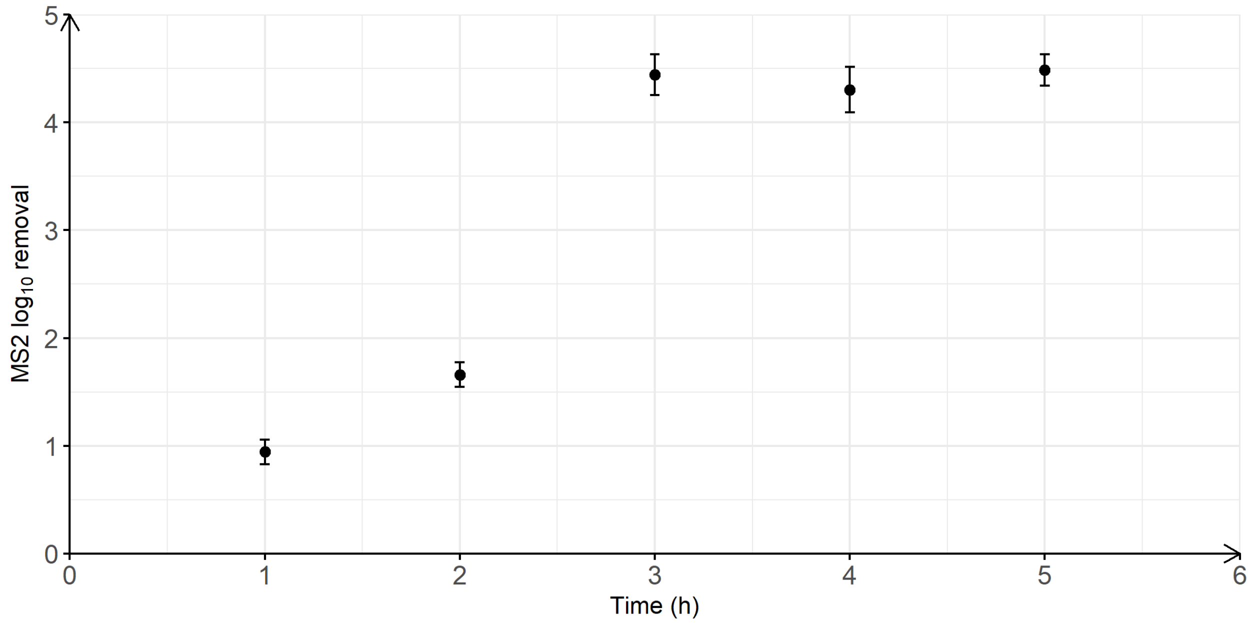

Figure S 2: MS2 log_{10} removal as a function of time. This batch experiment was performed at pH 7.7, with a mass of MWCNT equal to 15 mg, MS2 concentration of 10^6 PFU/mL, to evaluate the time of required to reach material saturation. One reactor was sampled at different times.

3. Description of the statistical model used to evaluate the effect of pH on MS2 adsorption on MWCNT

To evaluate the effect of the pH on MS2 LRV as a function of MWCNT mass, we ran a statistical model with Rstudio.

The hypothesis of this model was: if the pH affects MS2 LRV as a function of MWCNT mass, it would affect the intersect of the linear regression, because a net surface change would occur ether for MS2 or for MWCNT and consequently more or less MS2 could adsorb to the MWCNT for the same mass. However, the hypothesis further suggests that the change in LRV relative to the change in MWCNT concentration (slope) is unaffected at different pH, because the number of adsorption sites on MWCNT is proportional to MWCNT mass.

The model used was the following:

$$LRV = \beta_0 + \beta_1 MWCNT + \beta_2 I(pH7.7) + \beta_2 I(pH8.7)$$

pH5.2 data was used to plot the linear regression and I(pH=7.7) and I(pH=8.7) are switch functions either equal to 1 or 0.
